# Where have all the grouse ticks gone? Apparent decline in collections of *Haemaphysalis chordeilis* Packard

**DOI:** 10.1016/j.ijppaw.2022.11.007

**Published:** 2022-11-22

**Authors:** Andrea Egizi, Lauren P. Maestas

**Affiliations:** a Tick-borne Disease Program, Monmouth County Mosquito Control Division, 1901 Wayside Rd, Tinton Falls, New Jersey, 07724, USA; b Center for Vector Biology, Rutgers University, 180 Jones Ave, New Brunswick, NJ, 08901, USA; c USDA ARS – Cattle Fever Tick Research Laboratory, 22675 N. Moorefield Rd, Moore Air Base, Edinburg, TX, 78541, USA

**Keywords:** Ectoparasites, Conservation, Game birds, Population biology, Historical records

## Abstract

*Haemaphysalis chordeilis* Packard*,* also known as the grouse or bird tick, is a three-host tick native to North America. Literature from the early 20th century reported a widespread distribution of this tick across the US and Canada. As its name implies, ground-dwelling birds such as grouse and quail were frequent hosts, and occasionally large infestations were reported in domestic flocks making it a pest of economic importance. However, after the mid-1900's records of this species appear scarce, and a number of more recent studies of ticks on birds (including ostensibly favored host species) did not detect it. To confirm this perception with data, we conducted a literature search for collection records of this species and compared the records across two eras (pre-1965 and post-1965), finding very few records of *H. chordeilis* in recent years, despite increased attention brought to this genus by the detection of exotic *Haemaphysalis longicornis* Neumann populations in the eastern US. We also compiled a list of studies after 1965 that examined appropriate hosts for ectoparasites but failed to find *H. chordeilis*. We interpret the apparent decline of ticks in the context of documented population declines in several major host species over the same time frame and discuss whether ectoparasite populations should be subject to the same conservation consideration as their hosts.

## Highlights

• Grouse tick collections have declined precipitously since the 1960's.
• Declines in grouse tick collections may be related to declines in host populations.
• Their hosts include North American grassland birds of conservation concern.
• Conservation efforts can affect ectoparasite populations.

## Graphical abstract

**Figure:**
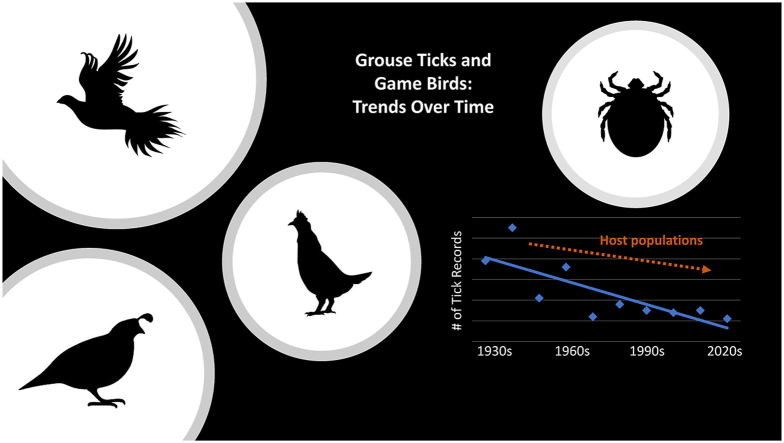

## 1 Introduction

Tick surveillance is an important tool for elucidating zoonotic disease cycles (Eisen and Paddock, 2021). Some tick species are easy to collect via tick drags/flags or passive surveillance of humans and/or domestic animals; others that specialize on wildlife hosts are only sampled by capturing and examining infested hosts. Collecting ticks from hosts can be costly, difficult and requires specialized expertise, permits, and registration of protocols to ensure humane treatment of animals (Cohnstaedt et al., 2012; Koser, 2019). Additionally, a great deal of sampling effort is required, as hosts are not uniformly infested (Wilson et al., 2002). These factors make it difficult to study cryptic-specialist species, and as a result, many aspects of their biology and life history are not well understood.

Despite this, many cryptic-specialist ticks are of direct or indirect medical and veterinary importance. They may serve as enzootic vectors and/or reservoirs for zoonotic pathogens. For example, *Ixodes affinis* Neumann, which is not known to bite humans, is nonetheless considered important for amplification of *Borrelia burgdorferi s.l.*, the agent of Lyme borreliosis, among wildlife reservoirs (Nadolny et al., 2011). Similarly, *Haemaphysalis leporispalustris* Packard, a rare parasite of humans, has been implicated in enzootic transmission of human pathogenic strains of *Rickettsia rickettsii,* the agent of Rocky Mountain spotted fever, in Brazil (Freitas et al., 2009). The winter tick, *Dermacentor albipictus* Packard, feeds primarily on wild cervids but is also a pest of domestic cattle, to which it can transmit *Anaplasma* spp. (Ewing et al., 1997).

The subject of this manuscript, *Haemaphysalis chordeilis*, was first described as *Ixodes chordeilis* by Packard in 1869. The type host was a Common Nighthawk (*Chordeiles minor* Forster) in Massachusetts, USA, from which its specific epithet was derived (Packard, 1869). *Haemaphysalis chordeilis* is closely related to *H. punctata* Canestrini and Fanzago and *H. cinnabarina* Koch, all members of subgenus *Aboimisalis* Dias*,* and early taxonomic uncertainty led to authors using variously one of the three names, or a combination thereof (e.g. *H. punctata cinnabarina*) for what is now considered to be unequivocally *H. chordeilis* (Guglielmone and Nava, 2014; Lindquist et al., 2016). For example, Nuttall and Warburton (1915) considered the three names to be synonymous, and authors such as Hewitt (1915) and Parker et al. (1932) used the name *H. cinnabarina* for ticks collected from North American game birds, while a specimen collected from cattle in Canada was called *H. punctata* in the Nuttall catalog (Keirans, 1985). However, both Hoogstraal (1973) and Barros-Battesti et al. (2008) reexamined type specimens of *H. cinnabarina* alongside specimens of *H. punctata* and *H. chordeilis* and concluded that the three are distinct species, separated by geography (*H. punctata* in Europe and southwestern Asia, *H. chordeilis* in North America, and *H. cinnabarina* known only from the Brazilian type).

*Haemaphysalis chordeilis* is thought to have a broad distribution throughout the United States and Canada (Lindquist et al., 2016). Very little is known about its natural history, such as behavior and phenology. One of the few studies collecting enough *H. chordeilis* to draw conclusions about phenology was conducted by Dick (1981), who reported that in Manitoba, Canada, *H. chordeilis* adults peaked on Sharp-tailed Grouse (*Tympanuchus phasianellus* Linnaeus) in early May, larvae in early July and nymphs in early August. According to Guglielmone et al. (2020), all stages of *H. chordeilis* commonly occur on ground-dwelling gamebirds (Galliformes: Phasianidae) and ground-foraging songbirds (Passeriformes: Icteridae) with several other avian groups serving as less frequent hosts of individual life stages. Mammals including humans are rare hosts (Guglielmone et al., 2014).

While not of direct medical importance, *H. chordeilis* has been implicated in transmission of *Francisella tularensis*, a zoonotic pathogen, to Sage Grouse (*Centrocercus urophasianus* Bonaparte) during an outbreak in Montana in 1931 (Parker et al., 1932). The bacterial agent of tularemia, *F. tularensis* was cultured from *“H. cinnabarina”* removed from a dead Sage Grouse, and guinea pigs injected with crushed ticks died of tularemia (Parker et al., 1932). Strangely, there is also a single case of fatal tick paralysis attributed to this species (the tick removed from the child's head was identified as *H. cinnabarina* by F. C. Bishopp) in British Columbia, Canada (Todd, 1919). However, the vector competence of *H. chordeilis* for *F. tularensis* has not been formally tested (Zellner and Huntley, 2019) nor has it been experimentally confirmed as a source of paralysis (Mans et al., 2004).

The early literature reports large infestations by *H. chordeilis* on game birds such as quail, turkeys and grouse (Bequaert, 1945; Bishopp and Trembley, 1945; Hadley, 1909; Nuttall and Warburton, 1915). However, most records of this species originate in the early 20th century and comparatively fewer papers and studies have reported them since. At the same time, many game bird populations have experienced declines due to loss/deterioration of habitat. (Sauer et al., 2013). Several historic hosts of *H. chordeilis* have experienced population declines since the 1960's; including the Northern Bobwhite (*Colinus virginianus* Linnaeus) (Hernández et al., 2013)*,* Sharp-tailed Grouse (Kirsch et al., 1973), and Ruffed Grouse (*Bonasa umbellus* Linnaeus) (Porter and Jarzyna, 2013). In this manuscript, we review published collection records of *H. chordeilis* to determine if reports of this species have declined over time and speculate on the influence of host declines on ectoparasite populations.

## 2 Methods

### 2.1 Literature review

We reviewed the literature on published collections of *H. chordeilis*, beginning with records included in major taxonomic and checklist publications such as Hooker et al. (1912), Nuttall and Warburton (1915), Bequaert (1945), Bishopp and Trembley (1945), Cooley (1946), Gregson (1956), Guglielmone et al. (2014), and Lindquist et al. (2016) and the references contained therein. We considered early identifications of “*H. cinnabarina*” or *“H. punctata”* within North America to be *H. chordeilis*, including 12 records of “*H. cinnabarina*” and 1 record of “*H. punctata*,” all dated prior to 1941. We next reviewed all publications listed for *H. chordeilis* in the “Index-Catalogue of Medical and Veterinary Zoology (ICMVZ), Special Publication No. 3: Ticks and Tickborne Diseases 1, Genera and Species of Ticks, Part 2: Genera H–N” (Doss et al., 1974). Lastly, we performed searches in Web of Science with the following search strings in February 2022: ALL = “*Haemaphysalis chordeilis”* (3 results), ALL = “*Haemaphysalis cinnabarina”* (1 result)*,* ALL = *“Haemaphysalis punctata”* AND ALL=(“North America” OR “United States” OR “Canada” OR “Mexico”) (6 results).

A list of collection records was made including data on the date, stage(s) collected, number of ticks, host, location, collector, accession numbers, and other pertinent notes (such as the taxonomic name indicated in the original record, or if the record was associated with a disease/fatality). To ensure there were no duplicate records, we sorted the dataset by date, location, and host, and removed observations with identical (or ambiguous) entries. For example, if a record did not give an exact year but was published in 1945, and we found a pre-1945 record with the same host and location, this was assumed to reference the same collection and the later (less precise) observation was excluded. In some cases, more specific collection details were found in later publications, and the additional reference was indicated in the ‘notes’ field.

For records without complete collection information, we made some educated deductions. For example, if a year was not given, then we used the year of the publication reporting it (as this is the latest it could have occurred). If the number of ticks collected was not reported, we gave these entries a value of 1 (assuming at least 1 tick was collected) except in cases where multiple stages were listed, for example records mentioning “nymphs and larvae” must have had at least 1 of each stage so these were given a value of 2. When reasonable assumptions were not possible, records lacking specific information were left out of analyses based on that information, for example, records that did not specify month of collection were excluded from analyses by month, and records that did not specify life stage were excluded from analyses by life stage.

We also attempted to assemble a list of publications that did not find *H. chordeilis* despite examining appropriate hosts within North America, with the following search strings in Web of Science: [ALL=(“ectoparasites” OR “ticks”) AND ALL=(“grouse” OR “pheasant” OR “quail” OR “wild turkey” OR “domestic turkey” OR “Phasianidae”) NOT ALL= (“ricinus” OR “sheep tick” OR “red grouse")] (59 results), [ALL=(“ectoparasites” OR “ticks”) AND ALL=(“meadowlarks” OR “blackbirds” OR “Icteridae”) NOT ALL= (“ricinus” OR “sheep tick” OR “red grouse")] (31 results), and [ALL=(“ectoparasites” OR “ticks”) AND ALL=(“sparrows” OR “Passerellidae” OR “Emberizidae”) NOT ALL= (“ricinus” OR “sheep tick” OR “red grouse")] (57 results). Searches were conducted in February 2022. The “NOT” search string addition was found necessary after a large portion of the results returned were from Europe. Any remaining results from other geographic locations were manually removed after inspection. To be included in the final negative dataset, the publication must have either reported all tick species found, or specifically stated that no ticks were found. More ambiguous publications (i.e. reporting counts of one species of tick without mentioning if other species were also found) were excluded.

### 2.2 New records

We acquired three adult specimens collected by T. D. Galloway in Manitoba, Canada which may represent new host records for this tick species. We report them here for inclusion in our analysis. The first specimen was a male removed from a Northern Goshawk (*Accipiter gentilis* Linnaeus) on Sept. 20, 2003. The second specimen is a female removed from a Red-tailed Hawk (*Buteo jamaicensis* Gmelin) on April 21, 2008. The third specimen is a female removed from a Northern Raven (*Corvus corax* Linnaeus) on April 15, 2021. Additional details for these collections are provided in Supplementary File 1 under “This manuscript.”

Morphological identification with established keys (Egizi et al., 2019; Keirans and Litwak, 1989; Lindquist et al., 2016) was confirmed by DNA barcoding. DNA was extracted in a non-destructive manner allowing the cuticle to remain intact for future morphological study as in Beati et al. (2012). Specimens were removed from ethanol and allowed to dry, then a small sliver of the posterolateral idiosoma was separated with a disposable microscalpel and both pieces placed in 180 μl Buffer ATL and 20 μl Proteinase K (Qiagen Inc., Valencia, CA) and lysed overnight at 56 °C. The following day, after vortexing to mix and centrifuging briefly to bring down condensation from the lids, the liquid was removed from each tube and extracted using a Qiagen DNeasy Blood and Tissue Kit following manufacturer's protocol, except for eluting in 50 μl of hot (72 °C) Buffer AE. The tick cuticle parts were returned to 2 mL cryovials with 95% EtOH for long term storage.

We amplified a 656 bp fragment of the mitochondrial DNA locus cytochrome oxidase I (*cox1*) with primers chelicerateFw (5′-TACTCTACTAATCATAAAGACATTGG-3′) and chelicerateRv (5′-CCTCCTCCTGAAGGGTCAAAAAATGA-3′) (Barrett and Hebert, 2005). This primer pair produced better sequence for *H. chordeilis* than the standard Folmer primers HCO2198/LCO1490 (Folmer et al., 1994). PCR reaction conditions were as follows: an initial activation step of 95 °C for 15 min, followed by 40 cycles of 95 °C for 30 s, 50 °C for 30 s, and 72 °C for 45 s; with a final extension of 72 °C for 7 min. The 25 μl reaction volume contained 12.5 μl AmpliTaq Gold™ 360 Master Mix (Life Technologies), 1 μl of each primer (10 μM), 1 μl template DNA, 1.6 μl MgCl_2_ (20 mM), 0.375 μl Bovine Serum Albumin (BSA; 10 mg/mL), and the remainder ultrapure water. PCR products were visualized on a 2% agarose electrophoresis gel, purified with ExoSAP-IT (USB Corporation, Cleveland, OH), and Sanger sequenced in both directions (Genewiz, South Plainfield, NJ). Sequences were assembled and trimmed in Geneious 10.2.3 (Kearse et al., 2012) and compared with known tick *cox1* sequences in NCBI BLASTn (Altschul et al., 1990) and BOLD identification system (Ratnasingham and Hebert, 2007) searches.

## 3 Results

### 3.1 Literature search

In total, we identified 158 published records of *H. chordeilis* collections, beginning with its original description (Packard, 1869) and ending with the last published collection in 2019 (Thompson et al., 2020) (T. Mather personal communication). Together with the three records published here, including one from 2021 (the most recent known collection at the time of this publication) the full dataset comprises 161 records (Supplementary File 1).

**Fig. 1:**
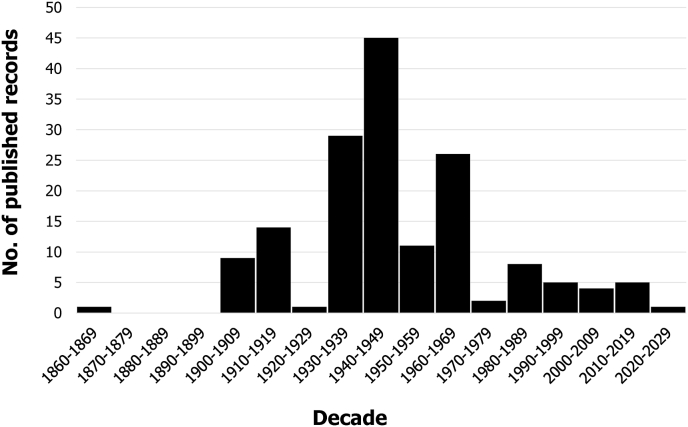
**Published records of *H. chordeilis* (N** = **161) by decade**. Note that when no year was given for the collection, the year of the publication was used, so some records may have occurred earlier.

The majority (82%) of *H. chordeilis* reports occurred pre-1965, with very few records attained between 1965 and 2021 (Fig. 1). The mean number of *H. chordeilis* reported per collection record was higher before 1965, although the confidence intervals overlap (mean: 27.9, 95% CI: −0.6-56.3 ticks before 1965, and mean: 2.9, 95% CI: −0.1-5.9 ticks after 1965). The sampling unit of each collection record ranged from a session of environmental collection (e.g. flagging/dragging), to ticks removed from one or more hosts of the same species. As explained earlier, collections from different host species, geographic locations, or dates were considered separate records.

**Fig. 2:**
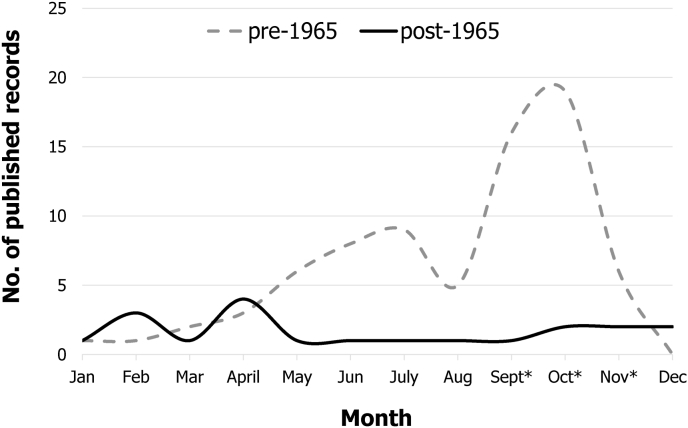
**Published records of *H. chordeilis* (N** = **96) by collection month**. Records that did not specify collection month are excluded. Asterisks denote autumn months where hunting season is open for most game birds in North America, which likely influenced collections of *H. chordeilis.*

Post-1965 tick collection records differ in several ways from the earlier records. Before 1965, 62.9% of tick collection records were from Galliform birds and 12.1% were from Passeriformes. After 1965, this pattern reversed: only 10.3% of records were from Galliformes and 62.1% were from Passeriformes. Additionally, there are no records of *H. chordeilis* attached to mammalian hosts post-1965 (Table 1). For records where a month of collection was given, most came from warmer months in the earlier collection period: between 1869 and 1964, 61.8% were collected April–September, while from 1965 to 2021, only 45.0% came from those months, and instead most collections were during cooler months (October–March) (Fig. 2).

**Table 1:** Count (%) of records by host type for various subsets of the data. Full = the complete dataset, 1869–2021; Pre-1965 = records dated 1964 and earlier; Post- 1965 = records dated 1965 and later; CAN only = records from Canada only; USA only = records from continental US only.

	Full	Pre-1965	Post-1965	CAN only	USA only
Galliformes	86 (53.4)	83 (62.9)	3 (10.3)	18 (41.9)	66 (61.1)
Passeriformes	34 (21.0)	16 (12.1)	18 (62.1)	3 (7.0)	27 (25.0)
Other Aves	8 (5.0)	5 (3.8)	3 (10.3)	2 (4.7)	5 (4.6)
Mammalia	17 (10.6)	17 (12.9)	0 (0.0)	12 (27.9)	2 (1.9)
Environment	8 (5.0)	6 (4.6)	2 (6.9)	4 (9.3)	4 (3.7)
Unknown	8 (5.0)	5 (3.8)	3 (10.3)	4 (9.3)	4 (3.7)

**Fig. 3:**
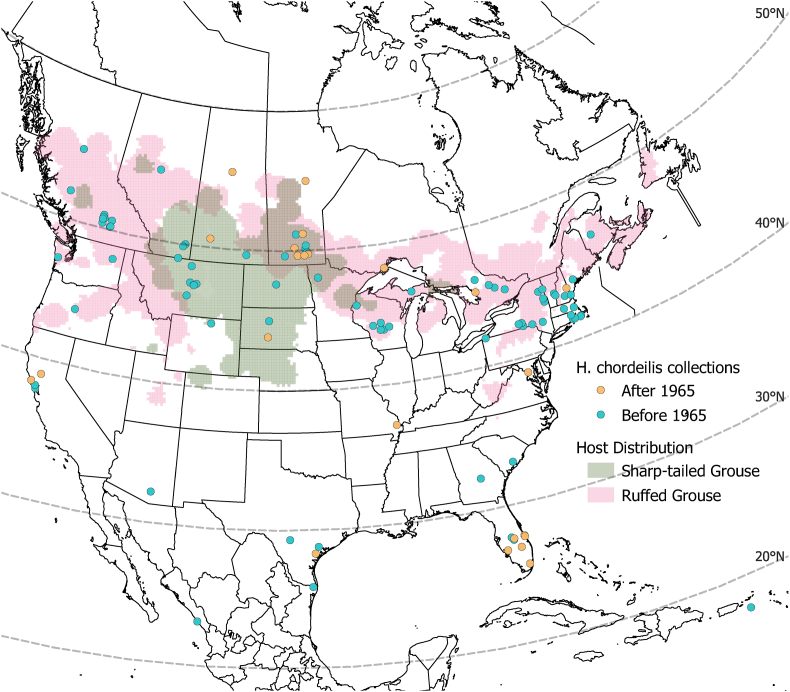
**Geographic locations of *H. chordeilis* records (N** = **152)**. Records with no location information are excluded. In cases where a specific location (town or site) was not given, the state or county midpoint was used instead, taken from https://www.mapdevelopers.com/geocode_tool.php. Base map developed by the North American Commission for Environmental Cooperation (CEC) and downloaded from https://www.sciencebase.gov/catalog/item/4fb555ebe4b04cb937751db9. Species range maps developed by the U.S. Geological Survey Patuxent Wildlife Research Center based on National Breeding Bird Survey data from 2011 to 2015 and downloaded from https://www.mbr-pwrc.usgs.gov/bbs/shape_ra15.html. Map created in QGIS Desktop v3.22.3.

Geographically, most of the records came from the continental United States (70.4%) and Canada (28.3%) although there was also one record each (0.66% each) from Sinaloa, Mexico (Hoffman, 1962) and the U.S. Virgin Islands (Beatty, 1944) (Fig. 3). These latter two records are outside of its normal distribution and may represent a misidentification or alternatively, stray ticks carried on a migratory bird. Within North America a majority of records (62%) occurred between 40 and 50° north latitude, possibly reflecting the distribution of favored host species (Fig. 3). The proportion of records from Canada increased slightly between the two time periods (26.8% pre-1965 to 34.5% post-1965). Of note, the majority of mammalian records came from Canada (Table 1), including 5/6 of the records on cattle and 3/4 of the records on humans (the 4th record did not have a location specified, but Bishopp and Trembley (1945) included it in a table of records from the United States Department of Agriculture, so it seems likely it was from the United States).

We identified 60 studies conducted in 1965 or later that failed to find *H. chordeilis* despite inspecting birds for ectoparasites (Supplementary File 2). Of these negative studies, 24 were either focused exclusively on Galliformes, such as Northern Bobwhite or included at least one Galliformes bird among a broader study. One survey of meadowlarks in South Texas during 1981–1982 (Teel et al., 1988) specifically noted the absence of *H. chordeilis* as specimens had been collected from meadowlarks in Texas previously (Nuttall and Warburton, 1915). Across the 60 negative studies, a minimum of 1134 Galliformes, 5656 Passeriformes: Icteridae, and 22,960 Passeriformes: Passerellidae were checked for ectoparasites (we say ‘minimum’ because some studies only reported birds with ticks, not the total number of birds inspected). These three host groups supported other tick species in 53/60 (88.3%) of the studies, most commonly *H. leporispalustris* (reported in 35/53 or 66.0% of the studies that found ticks) and *Ixodes scapularis* Say (found in 26/53 or 49.1% of the studies that found ticks).

### 3.2 New records

The *cox1* sequence for all three specimens was identical and has been uploaded to GenBank (NCBI Accession # OP787254). The top match in a BLASTn search was to *H. chordeilis* from Parry Sound, Ontario, Canada (98.72% identity, 95% coverage to MN991269.1 from Thompson et al., 2020), followed by several *H. punctata* sequences from Europe with 90–96% similarity.

These collections represent at least one new host record for *H. chordeilis* (Northern Raven) as our literature review did not previously detect any collections from this species. There are two prior records from Accipitridae, two males collected from a ‘marsh hawk’ reported by Bishopp and Trembley (1945) (likely referring to *Circus hudsonius* Linnaeus, now known as the Northern Harrier) and an unspecified number of females removed from an unidentified hawk species (simply listed as ‘hawk’) from Newport, Rhode Island, reported by Bequaert (1945). Depending on the identity of Bequaert's hawk, one or both of the Accipitridae records reported here (Red-tailed Hawk and Northern Goshawk) may represent a new host association for *H. chordeilis*.

## 4 Discussion

Based on our literature review, collections of *H. chordeilis* appear to have declined post-1965. While this could simply reflect changing trends in the timing and focus of tick collection surveys on birds, i.e. recent studies placing a greater emphasis on migratory passerines during spring and fall, we did identify at least a dozen studies post-1965 targeting Galliformes, most recently a study that surveyed 326 Wild Turkeys across Florida between 2000 and 2015 (Hertz et al., 2017) and did not detect *H. chordeilis* although they collected *H. leporispalustris*, *Amblyomma americanum* Linnaeus*,* and *I. scapularis*. Our count of ‘negative’ studies may in fact be an underestimate, as negative data (e.g., studies where no ticks are found) may be less likely to become published; to illustrate this point the authors were involved with unpublished tick collections from 74 Wild Turkeys in Delaware, where only *A. americanum* was collected (April–May 2019).

The failure to find *H. chordeilis* in recent surveys of game birds is intriguing due to the rather extreme language used to describe infestations in the early literature. For example, Bishopp and Trembley (1945) wrote “The writers have obtained information to the effect that the bird tick *often causes the death of poults* along the coast in Connecticut, and that young grouse reared and released in late summer on Fishers Island, N.Y., *often become heavily infested*, and that the mortality is rather high” (emphasis added). Nuttall and Warburton (1915) recorded that J.D. Mitchell, collecting ticks from hunted quail in Victoria County, TX in December 1907, stated “that there were 1000 ticks upon the 12 quail would be a conservative estimate.” It is difficult to reconcile these extremes with the scarcity of modern records, and how few ticks are recovered at a time, for example seven *H. chordeilis* recovered from 13 Florida Grasshopper Sparrows (*Ammodramus savannarum floridanus* Mearns) out of 163 birds surveyed between 2013 and 2015 (Hewett Ragheb et al., 2020).

Additionally, while the genus *Haemaphysalis* has been historically under-studied in North America, the discovery in 2017 of populations of the exotic Asian longhorned tick (*H. longicornis*) in the eastern US (Beard et al., 2018; Rainey et al., 2018) has accelerated searches for *Haemaphysalis* spp. on a wide array of host species. It seems unlikely that if *H. chordeilis* was as common as indicated historically, in areas such as New England, that it could have continued to remain undetected during this time.

It is possible that some of the pre-1960 studies could have misidentified *H. leporispalustris*, as despite being called the ‘rabbit tick’ this species is also frequently recorded from birds*.* Hooker et al. (1912) wrote of *H. chordeilis*: “They are very frequently associated on the hosts with the immature stages of *Haemaphysalis leporis-palustris*.” Certainly, immatures of the two species are difficult for non-experts to distinguish, however, many early records (including some of the most extreme infestations) included adult ticks, which are easier to discriminate. And many of those reports came from well-regarded taxonomists, decreasing the likelihood of misidentification. Additional supporting evidence comes from the Canadian National Collection of Insects, Arachnids and Nematodes, where specimens are curated by taxonomic experts, yet the same pattern emerges: 76/105 (72%) of *H. chordeilis* specimens in the collection were deposited prior to 1940 (Wayne Knee, Personal Communication).

A decline in *H. chordeilis* collections post-1965 is probably related to observed declines in primary host species over the same time frame (Hernández et al., 2013; Kirsch et al., 1973; Porter and Jarzyna, 2013). Conservation and management of game birds has become a priority, especially concerning species such as the Sage Grouse, threatened by habitat loss due to human settlements, herbicide use, agriculture (Braun, 1998), vector-borne disease (Kunkel et al., 2022) and oil and natural gas exploration in Western states (Green et al., 2017). The Northern Bobwhite provides another example of the loss of hosts for *H. chordeilis*, as fire suppression and a pathogenic landscape influence quickly disappearing quail populations (Brennan, 2015). In addition to game birds, several other commonly recorded hosts of *H. chordeilis* are grassland specialists, such as meadowlarks and grasshopper sparrows. For example, the Florida Grasshopper Sparrow, from which *H. chordeilis* has been recorded several times (Bishopp and Trembley, 1945; Delany et al., 1992; Delany and Forrester, 1997; Hewett Ragheb et al., 2020) is an endangered species that requires a specific type of xeric prairie habitat in south-central Florida (Hewett Ragheb et al., 2020).

Grassland birds are one of the most threatened bird groups in North America due to habitat loss and deterioration (Knopf, 1994; Sauer et al., 2013; Shaffer and DeLong, 2019). In the central and western US, agricultural intensification during the 20th century has eliminated or degraded much of the native prairie ecosystem. Practices such as overgrazing (continuous high densities of cattle on the same land) and fire suppression (preventing wildfire in an ecosystem meant to burn) can drastically alter grassland communities, promoting establishment of woody shrubs and invasive species and making the area less suitable for grassland specialists (Askins et al., 2007; Brennan and Kuvlesky Jr, 2005; Knopf, 1994). In the Eastern US, loss of grassland habitat is largely attributable to succession of former agricultural land into forests (Askins et al., 2007; Brennan and Kuvlesky Jr, 2005). This process also impacts the Ruffed Grouse, another frequent *H. chordeilis* host: though not a grassland species, it prefers forests of young successional age with high stem density (Dessecker and McAuley, 2001) and its decline in New York state has been linked to forest maturation (Porter and Jarzyna, 2013).

Decline and extinction of a host species can result in the corresponding loss of host-specific parasites, a phenomenon known as ‘coextinction’ (Stork and Lyal, 1993). Even if a host avoids extinction, conservation programs may intentionally remove their parasites (Harris et al., 2014) or the host population could fall below a threshold required to maintain parasite transmission (De Castro and Bolker, 2005). At least 63 species of hard ticks are at risk of extinction along with their threatened or endangered host species, including *Dermacentor rhinocerinus* Denny (a parasite of endangered African rhinoceroses) and *Amblyomma tuberculatum* Marx (the gopher tortoise tick) in the southeastern US (Durden and Keirans, 1996; Mihalca et al., 2011). Although historically overlooked, the argument that parasites should be subject to the same conservation considerations as their hosts has been gaining momentum, and a comprehensive plan to address threats to parasite biodiversity was recently authored (Carlson et al., 2020).

In addition to host population declines, *H. chordeilis* populations could have been affected by tick-specific pathogens or parasites, such as the parasitoid wasp *Ixodiphagus texanus* Howard. While *I. texanus* has not yet been reported from *H. chordeilis*, possibly due to lack of study, it has been reported from field-collected *H. leporispalustris* ranging from Texas to Canada (Davis and Campbell, 1979; Hu et al., 1998). Because *H. chordeilis* and *H. leporispalustris* are described as frequently overlapping on the same hosts in early literature (e.g., Hooker et al., 1912), it is possible that the parasitoid could have ‘spilled over’ from *H. leporispalustris* to affect *H. chordeilis*. When an interaction between two species is indirect and mediated by a shared enemy, such as a predator or parasitoid, this is known as enemy-mediated apparent competition (Chaneton and Bonsall, 2000). It is possible that the presence of *H. leporispalustris,* as a more suitable host for the parasitoid, had a negative impact on less-suitable H. chordeilis (e.g., Heimpel et al., 2003).

An intriguing finding of our analysis is that most records of *H. chordeilis* from mammals occurred in Canada, specifically central/western Canada (Manitoba, British Columbia, and Saskatchewan) and before 1965 (Fig. 3). One potential explanation is the local establishment in North America of Eurasian *H. punctata*, as posited by Hadwen (1910). In 1909, Hadwen was given two female ticks removed from a steer in Winnipeg, Manitoba, which he subsequently sent to eminent parasitologist G. H. F. Nuttall at the University of Cambridge, who identified them as the red sheep tick, *Haemaphysalis punctata* (Hadwen, 1910). However, these specimens were later reexamined and identified as *H. chordeilis* by Keirans (1985). *Haemaphysalis punctata* is a well-known parasite of mammals, especially cattle (Guglielmone et al., 2020), and its establishment in Manitoba could explain the geographic distribution of mammalian records we observed. However, several details are unclear from Hadwen's report, including the travel history of the cattle in question. While occasional introductions of *H. punctata* to North America are a possibility (Tufts and Diuk-Wasser, 2021), an alternative hypothesis is that North American *H. chordeilis* encompasses multiple endemic forms or potentially subspecies, with varying host specificity. Possible support for this second hypothesis comes from Bequaert (1945) who noted morphological variation (difference in the length of the scutum compared to its width) between specimens of *H. chordeilis* from Massachusetts and Canada vs. specimens from Texas. Bishopp and Trembley (1945) considered *H. chordeilis* to have an eastern distribution in North America, with western records attributed to *H. cinnabarina*, although they do not explain the reasons for this distinction. Of note, *cox1* sequences from *H. chordeilis* collected in western and eastern Canada (Manitoba vs. Parry Sound, Ontario) shared 98.85% identity (see Results). Further phylogeographic study of *H. chordeilis* is needed to resolve these questions, however, the rarity of specimens makes this a challenging pursuit.

## 6 Conclusions

*Haemaphysalis chordeilis* collection records have become scarcer in recent decades, possibly reflecting population declines in this tick species. Declines in a parasite can be caused by reductions in host populations. In the case of *H. chordeilis*, they may be attributable to reductions in grassland and game bird populations that have occurred in North America over the last half-century. As conservation and management efforts intensify for these bird species, it is possible that *H. chordeilis* populations could rebound in the future; alternatively, if ectoparasite biodiversity is neglected, they could disappear entirely.

## Funding

This research did not receive any specific grant from funding agencies in the public, commercial, or not-for-profit sectors.

## Declaration of competing interest

The authors declare that they have no known competing financial interests or personal relationships that could have appeared to influence the work reported in this paper.
